# Exploring the Relationship between Obesity, Metabolic Syndrome and Neuroendocrine Neoplasms

**DOI:** 10.3390/metabo12111150

**Published:** 2022-11-21

**Authors:** Xiaoyang Lan, Nicola Fazio, Omar Abdel-Rahman

**Affiliations:** 1 Department of Medicine, University of Alberta, Edmonton, AB T6G 1Z2, Canada; 2 Division of Gastrointestinal Medical Oncology, European Institute of Oncology (Istituto Europeo di Oncologia—IEO), Istituto di Ricovero e Cura a Carattere Scientifico—IRCCS, 20141 Milan, Italy; 3 Department of Oncology, Cross Cancer Institute, University of Alberta, Edmonton, AB T6G 1Z2, Canada

**Keywords:** obesity, neuroendocrine neoplasm, body mass index, nutrition

## Abstract

Obesity is a major burden for modern medicine, with many links to negative health outcomes, including the increased incidence of certain cancer types. Interestingly, some studies have supported the concept of an “Obesity Paradox”, where some cancer patients living with obesity have been shown to have a better prognosis than non-obese patients. Neuroendocrine neoplasms (NENs) are malignancies originating from neuroendocrine cells, in some cases retaining important functional properties with consequences for metabolism and nutritional status. In this review, we summarize the existing evidence demonstrating that obesity is both a risk factor for developing NENs as well as a good prognostic factor. We further identify the limitations of existing studies and further avenues of research that will be necessary to optimize the metabolic and nutritional status of patients living with NENs to ensure improved outcomes.

## 1. Obesity and Human Health

Obesity, defined as a body mass index (BMI) of equal to or greater than 30, has been linked to increased rates of coronary artery disease, type 2 diabetes mellitus (T2DM), cancer, and mental health disorders [1,2,3]. Multiple studies have demonstrated an association between obesity and all-cause mortality, with one study estimating that increased BMIs accounted for 4.0 million deaths worldwide in 2015 [4,5]. The simplicity of BMI as a metric makes it a useful screening tool for obesity. However, BMI is known to be an imperfect metric, with some groups developing more advanced measures of body composition and adiposity that are more predictive of metabolic risk [6].

Obesity is a component of a cluster of metabolic disturbances that have been termed metabolic syndrome (MS). While various definitions for MS exist, the National Cholesterol Education Program’s Adult Treatment Panel III (NCEP: ATP III) definition is easily applicable to clinical practice. The NCEP: ATP III panel defines MS as three or more of: central obesity (waist circumference of >102 cm in males or >88 cm in females), hypertriglyceridemia (triglycerides of ≥1.7 mmol/L), low high-density lipoprotein-cholesterol (HDL-C of <1.0 mmol/L in males or <1.3 mmol/L in females), hypertension (blood pressure of ≥135/85 or on medication), and a fasting plasma glucose of ≥6.1 mmol/L [7,8]. MS has also been identified to be an independent risk factor for the development of breast, bladder, and gastrointestinal malignancies [9].

This review article will summarize the existing evidence that obesity and metabolic syndrome directly impact cancer incidence and outcomes, with a particular focus on neuroendocrine neoplasms (NENs).

## 2. The Impact of Obesity on Cancer Risk and Outcomes

Obesity has been correlated with both increased cancer risks and poorer cancer outcomes in a number of studies [10,11]. In a prospective cohort study of more than 900,000 adults in the United States of America (USA), a BMI of at least 40 was associated with a greater risk of death from all cancers, with relative risks of 1.52 and 1.62 for men and women, respectively [11]. In 2016, the International Agency for Cancer Research performed a review of the literature and concluded that sufficient evidence exists for a preventative effect of the absence of excess body fat on the risk of developing cancers of the gastrointestinal tract, breast and ovary, as well as renal cell carcinoma, meningioma, thyroid cancer and multiple myeloma [12]. The proposed mechanisms for the impact of obesity on cancer risk include its association with insulin resistance, high levels of insulin-like growth factors, the production of endogenous sex steroids and a state of chronic inflammation [13,14].

Beyond having an impact on cancer risk, other studies have asked whether weight loss interventions can affect the outcomes of individuals already diagnosed with cancer. A recent systematic review specifically examined the effects of weight loss interventions on mortality, cardiovascular disease and cancer [15]. While high quality evidence from 34 trials demonstrates that weight loss interventions can reduce all-cause mortality, very low quality evidence supports a specific benefit on cancer-related mortality (risk ratio 0.58, 95% confidence interval (CI) 0.30 to 1.11) [15].

The effects of weight changes in cancer outcomes is further complicated by cancer cachexia, a phenomenon of weight loss, anorexia and muscle wasting that has been implicated in the mortality of at least 20% of all patients afflicted with cancer [16,17,18]. While multiple definitions for cancer cachexia exist, a commonly used set of criteria decided through international consensus includes weight loss of >5% over a 6 month period in the absence of starvation, a BMI of <20 and any degree of weight loss of >2%, or sarcopenia as measured with appendicular skeletal muscle index (SMI; male < 7.26 kg/m^2^ and female < 5.45 kg/m^2^) and any degree of weight loss of >2% [18,19]. Importantly, interventions designed to address the nutritional needs of patients with cancer cachexia have demonstrated improvements in survival [20]. In a retrospective review by Gannavarapu et. al. of 3180 patients with thoracic or gastrointestinal malignancies [21], pre-treatment cancer-associated weight loss was identified in 34% of patients at diagnosis, and it was associated with reduced survival (hazard ratio (HR) 1.26, 95% CI 1.13 to 1.39). Weight loss during cancer treatment may also independently limit the dose of systemic therapies that patients receive while increasing the likelihood of toxicities [22]. While some limited evidence is available to support a beneficial impact of weight loss on cancer-specific outcomes, a holistic approach designed to meet the specific nutritional requirements of each cancer patient, rather than simply targeting a specific BMI level, is warranted in the management of advanced cancers.

Interestingly, some studies have actually observed improved outcomes in patients with obesity and cancer [23], a phenomenon that has been termed the obesity paradox [24]. This effect has been observed in cancers of the lung [25], kidney [26,27], breast [28] and colon [29], as well as hematologic malignancies [30,31]. In a large meta-analysis of 203 cancer studies performed by Petrelli et. al. [10], obesity was associated with reduced survival and increased risk of recurrence, with the notable exception of lung cancer, renal cell carcinoma and melanoma, where patients with obesity had better survival outcomes [10]. Several possible explanations exist for the obesity paradox, including issues that have been raised around experimental design and interpretation. Exposure to selection bias, the timing of when BMI is calculated and the existence of confounders that can decrease BMI such as cigarette smoking can make the studies challenging to interpret [24]. Cancer cachexia is well-established as a poor prognostic marker which can specifically impact the validity of post-diagnosis BMI as a metric [32,33]. The use of post-diagnosis BMI can also lead to reverse causation, where significant weight loss can be the result of advanced cancer, obscuring the impact of pre-diagnosis BMI on cancer outcome [24]. The use of pre-diagnosis BMI, or serial measurements of weight during disease course, may help address these concerns. Other groups have also argued that BMI alone is not an adequate measure of obesity [34,35] as it is significantly influenced by factors such as age and sex [36] and does not take into account the difference between visceral and subcutaneous fat [37,38].

Regardless of the uncertainties surrounding the obesity paradox, one potential contributor for the positive impact of obesity on cancer survival is its influence on specific treatments. While obesity can be associated with more surgical complications [39] and negative impacts on chemotherapy efficacy [40], the chronic inflammatory state associated with obesity has been hypothesized to improve the efficacy of checkpoint inhibitors and other forms of cancer immunotherapy [41,42]. The validity of the obesity paradox in cancer remains controversial in the literature, and further research into the area will be necessary to define the potentially protective role of obesity in cancer mortality.

## 3. Neuroendocrine Neoplasms

NENs comprise a group of malignancies that originate from the neuroendocrine cells of a diversity of primary sites including the gastrointestinal tract, respiratory tract, larynx, central nervous system, thyroid, kidneys and urogenital system [43,44]. NENs generally arise sporadically, although they can rarely be associated with multiple endocrine neoplasia type 1 or other heritable cancer syndromes [45]. NENs may be broadly subdivided into well-differentiated neuroendocrine tumors (NETs) and poorly differentiated neuroendocrine carcinomas (NECs) [45]. The majority of NETs immunohistochemically express the typical neuroendocrine markers chromogranin A and synaptophysin [43]. Some NETs are considered functional, retaining the ability to secrete hormones such as gastrin, insulin, glucagon and vasoactive intestinal peptide (VIP), thereby resulting in characteristic clinical syndromes [46]. NETs may also retain the characteristic ability of neuroendocrine cells to produce and secrete the amine serotonin, which can result in a characteristic syndrome of diarrhea, peripheral vasomotor symptoms, bronchoconstriction and carcinoid heart disease, known as carcinoid syndrome (CS) [43,47,48]. However, the older terminology of “Carcinoid” tumor for gastroneteropancreatic (GEP) NETs has fallen out of favor as NETs have come to represent true malignancies [49]. In contrast to well-differentiated NETs, neuroendocrine carcinomas (NECs) are poorly differentiated tumors that express fewer neuroendocrine markers, have higher rates of nuclear atypia and proliferation and are associated with poorer overall outcomes [44].

The prognosis and management of NENs is dependent on primary tumor site, histological grade and tumor, node and metastasis (TNM) staging [50]. Gastroenteropancreatic NENs are graded based on the Ki-67 index and mitosis [45]. TNM staging systems exist for GEP, lung and thymic NETs [45,51]. The overall prognosis for NETs is relatively favorable, with 5-year survival rates in the range of 60–80% [47,50,52,53].

## 4. Obesity, Metabolic Syndrome, and Incidence of Neuroendocrine Tumors

There has been an increase in the reported prevalence of NENs over time, explained at least in part by improvements in cancer screening and NEN classification [47,52,54,55]. In a recent analysis of the Surveillance, Epidemiology, and End Results (SEER) program that identified 65,971 cases of NETs in the USA between 1973 and 2012, the age-adjusted incidence rate was found to have increased 6.4-fold to 6.98 per 100,000 individuals, with the most common sites being the lung and the gastrointestinal tract [55]. Changes in demographic and environmental factors may play an important role in the increased prevalence of NENs, as well. Specifically, several lines of evidence have pointed toward obesity and MS as risk factors for NETs (Table 1) [56,57,58,59,60,61]. For example, a 2016 meta-analysis of 24 studies identified elevated BMI and diabetes as the second most relevant risk factor for NENs of the stomach, pancreas and small intestine after family history [59]. A USA-based case-control study of 740 patients with NETs also identified diabetes mellitus as a significant risk factor of gastric NETs, with a particularly strong effect in women [60]. In a single-center case-control study comparison of 96 individuals with well-differentiated GEP- NETs and 96 matched controls from the general population, a statistically significant association was observed between GEP-NETs and MS, as well as individual factors such as waist circumference, fasting triglycerides and fasting plasma glucose [61]. A follow-up study from the same group further described that patients with MS and GEP-NETs are more likely to present either with lower grade tumors or at an advanced stage [62]. Taken together, multiple studies support a link between MS or its individual components and a risk of developing NETs of multiple tissues of origin.

Beyond the studies supporting MS as a risk factor for NENs, obesity itself seems to be an independent risk factor for NENs. Interestingly, there have been multiple observations of increased incidences of gastric NETs identified during routine endoscopic evaluation prior to bariatric surgery [63,64,65,66,67]. When extrapolated, these studies suggest that the incidence of gastric NETs ranges from 0.23–0.358% in obese patients in comparison to 0.001–0.002 % in the general population [63,64]. Mechanistically, this may be explained by the increased gastric atrophy and G-cell hyperplasia associated with type 1 gastric NETs [64]. The colonization of H. pylori has been linked to the incidence of gastric NETs through the induction of multiple signaling pathways that lead to atrophic gastritis and the hyperplasia of enterochromaffin-like cells [68]. Additionally, the appendix has been identified as a preferential site of gastrointestinal NETs when an appendectomy is performed along with a bariatric procedure [69].

Several studies have directly examined the influence of BMI on the risk of developing NENs. In the study by Santos et. al. [61], visceral obesity, defined as a waist circumference of >80 cm for females and >94 cm for males, was reported as a risk factor for well-differentiated GEP-NETs (OR 2.5, 1.4–4.6). Leoncini et. al. [59] also performed a meta-analysis of risk factors for NENs where two of the three case-control studies demonstrated an association of BMI with pancreatic NETs, with an adjusted summary effect estimate of 1.37 (95% CI 0.25–7.69, *p* < 0.001), although the data for the small intestine and rectum were inconclusive. Conversely, a study by Hassan et. al. [60] actually demonstrated a 60–70% reduction in the risk of developing pancreatic and small bowel NETs in overweight and obese individuals. Further complicating this matter, complex interactions exist between metabolism, the microbiome and the risk of developing NENs. Interestingly, a link has been established between inflammatory bowel disease (IBD) and NENs, and it may be the result of associated changes in the gut microbiome [68,70]. Overall, evidence exists for obesity and MS as independent risk factors for the development of NENs, although further studies are necessary to reconcile some of the controversial data in the literature and identify whether this relationship is exclusive to NENs of certain primary sites.

Despite the observations that obesity and MS are risk factors for NENs, it is also established that NENs are associated with changes in nutritional status that can lead to weight gain. Indeed, patients with GEP-NETs have been demonstrated to have a poorer overall nutritional status in comparison to the general population, including less frequent adherence to a Mediterranean diet and increased consumption of simple carbohydrates and polyunsaturated fats [71]. In certain instances, weight gain can also be a biological consequence of functional NENs rather than a risk factor for tumor initiation. Firstly, the ectopic secretion of adrenocorticotropic hormone (ACTH) from pulmonary NETs has been described, resulting in Cushing’s syndrome and unintentional weight gain [72,73,74]. In one case series of 918 GEP-NETs and thoracic NETs, the prevalence of ectopic ACTH secretion was reported to be 3.2% and associated with poorer patient survival [75]. In these cases, definitive therapy such as surgical resection can result in weight loss, although specific therapy for hypercortisolemia such as Metyrapone can also be used [72,74]. Secondly, insulinomas are rare tumors that may occur sporadically or in association with multiple endocrine neoplasia type 1 (MEN1) syndrome, and they can also manifest with weight gain as patients attempt to relieve hypoglycemic symptoms by excess food intake [76,77,78]. Thirdly, the secretion of ghrelin from NETs may also act to maintain BMI in patients with metastatic disease and counteract the effects of cancer cachexia [79,80]. Lastly, an especially devastating condition known as rapid-onset obesity with hypoventilation, hypothalamic and autonomic dysregulation (ROHHAD) has also been associated with NETs (ROHHADNET) [81]. ROHHAD has been reported exclusively in the pediatric population, often presents initially as rapid weight gain and can be quickly fatal due to the impairment of the central respiratory drive [81,82]. ROHHADNET patients typically present with tumors of neural crest origin, such as ganglioneuromas [81,83]. Future studies that examine the correlation between obesity and non-functional NENs may help to determine whether the relationship is causal rather than a reflection of the underlying metabolic changes induced by NENs.

**Table 1:** Summary of the evidence linking obesity and metabolic syndrome to increased incidences of neuroendocrine tumors.

Citation	Study Population	Findings
Hassan (2008) [60]	Retrospective study of 740 patients with NETs and 924 healthy controls	In men, overweight individuals had a reduction in the risk of developing gastric, small bowel, pancreatic and lung NETs. In women, overweight individuals had a reduction in the risk of small bowel NETs. A long-term history of diabetes is a risk factor for gastric NETs (AOR 5.6, 2.1–14.5), particularly in women (AOR 8.4, 95% CI 1.9–38.1).
Mottin (2009) [63]	Retrospective study of 8383 patients who had bariatric surgery for morbid obesity from 2000–2007	Incidence of carcinoid tumors is estimated to be 358 per 100,000 in obese people compared to 1–2 per 100,000 people in the general population.
Capurso (2009) [84]	Case-control study of 162 pancreatic neuroendocrine tumors and 648 controls	A recent diagnosis of diabetes (≤12 months) is an independent risk factor for pancreatic NETs (OR 40.1, 95% CI 4.8–328.9). No differences in the mean BMI were observed between the cases and controls.
Crea (2011) [69]	Retrospective study of 588 patients who had bariatric surgery, 477 of which underwent routine appendectomies	Seven patients were identified with appendiceal carcinoid tumors (1.4%).
Cross (2013) [66]	Retrospective study of 237 small intestinal cancers, including 124 malignant carcinoid tumors, from the National Institutes of Health and the American Association of Retired Persons (NIH-AARP) Diet and Health Study	Increased risks of malignant carcinoid tumors of the small intestine were observed in those with a BMI of ≥35 kg/m^2^ compared to those with a BMI of 18.5 to < 25 kg/m^2^ (HR 1.95, 95% CI 1.06–3.58).
Zhan (2013) [67]	Case-control study of 196 patients with insulinoma and 233 controls	BMIs were higher in the patients with insulinoma compared to the controls (27.42 ± 4.54 compared to 23.59 ± 3.21, *p* < 0.0001); however, this was not significant in the multivariate analysis.
Halfdanarson (2014) [85]	Case-control study of 355 patients with pancreatic neuroendocrine tumors and 602 controls	Diabetes was more common in the patients with pancreatic neuroendocrine tumors compared to the controls (19% vs. 11%, *p* < 0.001).
Jung (2014) [58]	Cross-sectional study of 57,819 patients who underwent screening colonoscopy, of which 101 were diagnosed with rectal neuroendocrine tumors	Low HDL was an independent risk factor for rectal NETs (adjusted OR 1.85, 95% CI 1.10–3.11). Metabolic syndrome, high triglycerides and insulin resistance were more common in the patients with rectal NETs in the univariate analysis, although these were not independent risk factors.
Santos (2018) [61]	Case-control study of 96 patients with well-differentiated GEP-NETs and 96 matched controls	The presence of metabolic syndrome was associated with an increased risk of developing GEP-NETs (*p* = 0.003), with the risk increasing by the number of metabolic syndrome components (OR 3.40, 95% CI 1.17–9.86, *p* = 0.024 for four components and OR 5.15, 95% CI 1.15–23.01, *p* = 0.032 for five components).
Feola (2021) [57]	Retrospective case-control study of 148 patients with sporadic gastroenteropancreatic neuroendocrine neoplasms and 210 controls	The independent risk factors for GEP-NENs include T2DM (OR 2.5, 95% CI 3.9–4.51, *p* = 0.002) and obesity (OR 1.88, 95% CI 1.18–2.99, *p* = 0.007). Metformin is a protective factor in patients with T2DM (OR 0.28, 95% CI 0.08–0.93, *p* = 0.049). T2DM is associated with more advanced (OR 2.39, 95% CI 1.05–5.46, *p* = 0.035) and progressive disease (OR 2.47, 95% CI 1.08–5.34, *p* = 0.03).

## 5. Obesity and Patient Outcomes in NENs

While the above studies support obesity as a risk factor for NENs oncogenesis, early evidence has actually pointed toward a protective effect of increased BMI for patients already diagnosed with NENs (Table 2). In a recent analysis by our group of 1010 patients with NENs, a positive correlation was observed between survival outcomes and increasing BMI [86]. Indeed, the best outcomes were seen in the 30.6% patients categorized as obese (BMI of ≥30 kg/m^2^), and an underweight BMI was associated with poorer survival (HR 1.74; 95% CI 1.11–2.73) [86]. The effect was also preserved when BMI was used as a continuous variable (HR with increasing BMI: 0.97; 95% CI: 0.95–0.98) [86]. The protective effect of obesity on survival was maintained in independent analyses of 611 patients with NETs and 399 patients with NECs [86]. An important limitation of this study is that only BMI at diagnosis was available as a metric. As BMI is subject to change, longitudinal measurements of BMI over disease course may provide more information about its impact on outcomes. In a survey of 355 NET patients, Pape et. al. [87] described that 36% of patients had already experienced weight loss at the time of diagnosis and cancer cachexia was a significant contributor to mortality. This is supportive of decreased BMI as a poor prognostic indicator for NETs in our study, highlighting the importance of the targeted management of cachexia to improve patient outcomes. Importantly, the prevalence of cancer cachexia in NENs is not well established, although it is thought to be moderate given the generally slow-growing nature of well-differentiated NENs and good overall outcomes [88,89].

Interestingly, the presence of MS was also previously observed to correlate with both low-grade and disseminated or metastatic GEP-NETs [62]. Nevertheless, a stratified analyses of patients with different tumor stages preserved the protective effect of increasing BMI in our study [86]. Overall, the important limitations of our study include the use of BMI (an imperfect correlate of nutritional status and visceral obesity), lack of longitudinal weight data and the unavailability of information on other prognostic factors such as performance status and received treatments [86].

Several additional studies have delved into the potentially protective effect of increased BMI on NEN outcomes. A study of 324 patients with pancreatic NETs confirmed that a BMI of <20 was a negative prognostic factor, although the effect was not preserved in a multivariate analysis [90]. In a different study that examined 128 non-functioning pancreatic NETs, a BMI of ≥25 was not associated with differences in metastases or overall survival, although a comparison was not made with a BMI of ≥30 group [91]. A focused analysis of 22,096 patients diagnosed with GEP-NETs within an inpatient setting demonstrated a decreased likelihood of inpatient mortality in obese patients (OR 0.6, multivariate *p* = 0.02) and an increased likelihood of inpatient mortality in patients suffering from malnutrition [92]. This study was only able to examine all-cause mortality, and it was further limited by the use of *The International Classification of Diseases, Ninth Revision* (ICD-9) codes for weight status rather than BMI or other biometric measurements [92]. While some existing evidence points toward improved short- and long-term survival in obese patients that are diagnosed with NENs, further analyses are necessary to define this relationship and identify the impact of obesity on the NENs of different tumor sites. Importantly, the impact of BMI and nutritional interventions on the survival of patients with NENs has not been evaluated in clinical trials or prospective studies, making it difficult to establish a direct role for obesity’s effects on patient outcomes. A majority of the current literature has also focused on gastrointestinal NETs, although our analysis demonstrated that obesity is a protective factor for NENs with extra-gastrointestinal system primary sites and NECs, as well [86].

One potential explanation for the obesity paradox in NENs is the impact of BMI on the response to cancer treatment, with likely different impacts depending on the specific modality of therapy. In a study that examined 30 patients with metastatic NETs, improved survival in response to everolimus was observed in patients with higher SMIs and BMIs, although the comparison was only made between patients with a BMI of <18.49 and BMIs ranging from 18.49–24.99 [93]. This may be reflective of the poor outcomes of sarcopenic patients or possibly an effect of increased visceral adiposity on tumor responsiveness to mammalian target of rapamycin (mTOR) inhibition [93]. In a study of 67 patients with liver metastases undergoing chemoembolization, a linear relationship was also observed between BMI and tumor responsiveness [94]. Conversely, in a study of 19 patients with metastatic NEC that were receiving platinum-based chemotherapy, a BMI of ≥25 was actually associated with poorer survival outcome (PFS of 19.3 months and 6.2 months in the BMI < 25 and BMI ≥ 25 groups, respectively, with *p* = 0.006) [95]. Therefore, both positive and negative associations between BMI and treatment response have been observed in NENs, which is likely reflective of the complex interactions between tumor biology, the microenvironment and mechanisms of various treatments. Further studies in obesity and NEN outcomes should stratify patients based on the specific treatments received to clarify whether the relationship reflects the underlying biology of the disease or the interaction of specific treatments with the metabolic changes observed in obesity.

**Table 2:** Summary of the evidence examining the link between BMI and metabolic syndrome with a prognosis of neuroendocrine tumors.

Citation	Study Population	Findings
Marrache (2007) [94]	Retrospective study of 67 patients with liver metastases of endocrine tumors treated with transcatheter arterial chemoembolisation	Increasing BMI was significantly associated with tumor responsiveness to TACE (OR 1.3, 95% CI 1.04–1.63, *p* = 0.022) and delayed time to progression (HR 0.85, 95% CI 0.76–0.86, *p* = 0.01).
Ekeblad (2008) [90]	Retrospective study of 324 patients with pancreatic endocrine tumors	Patients underweight at diagnosis (a BMI of <20 kg/m^2^) had a poorer prognosis (HR 2.5, *p* = 0.005). This effect was not retained in a multivariate analysis.
Cherenfant (2013) [91]	Retrospective study of 128 patients with non-functioning pancreatic neuroendocrine tumors	No association was seen between BMI and the risk of distant metastasis or death.
Glazer (2014) [92]	Retrospective study of 22,096 patients discharged from hospital with abdominal neuroendocrine tumors	Obesity was associated with decreased rates of inpatient mortality in patients with NET (OR 0.6, multivariate *p* = 0.02), and malnutrition was associated with higher rates of mortality (9% vs. 2%, multivariate *p* < 0.0005). The rate of inpatient hospital complications was similar between obese and non-obese patients, but it was increased in malnourished individuals (15% vs. 10%, *p* < 0.0005).
Bongiovanni (2015) [95]	Retrospective study of 19 patients with metastatic gastroenteropancreatic neuroendocrine carcinoma treated with cisplatin or cisplatin/etoposide	Patients with lower BMIs had better overall survival and progression-free survival than patients with BMIs of ≥25. The mOS in the lower BMI group was not reached. The BMI ≥ 25 group had an mOS of 11.7 months (95% CI 5.6–13.5, *p* = 0.029).
Santos (2019) [62]	Prospective study of 134 patients with well-differentiated gastro-entero-pancreatic neuroendocrine tumors	There was an increased likelihood of metabolic syndrome in patients with a well-differentiated GEP-NET of grade G1 (OR 4.35, 1.30–14.53) and disseminated disease (OR 4.52, 95% CI 1.44–14.15).
Abdel-Rahman (2022) [86]	Retrospective study of 1010 patients with NENs of any primary site between 2004–2019, with complete BMI information	Patients with obesity (a BMI of >30 kg/m^2^) had the best survival outcomes, while underweight status was associated with poorer survival. These results were maintained on a stratified analysis by histology (NEC or NET), tumor stage, and primary site. The overall hazard ratios (OHR) were 0.60 (0.47–0.75) for obese individuals and 1.74 for underweight individuals (1.11–2.73).
Ranallo (2022) [93]	Retrospective study of 30 patients with well-differentiated, metastatic neuroendocrine tumors treated with everolimus	The median progression-free survival was lower in underweight patients (BMI of ≤18.49, mean PFS 3.2 months, 95% CI 0.9–6.7) compared to normal weight patients (mean PFS 10.1 months, 95% CI 3.7–28.4 months), with *p* = 0.011.

## 6. Diabetes, Obesity and NENs

Multiple studies have pointed towards increased BMI as the single most important risk factor for the development of T2DM [3,85,96,97,98]. It is therefore important to determine how diabetes and NENs interact independent of obesity. It is hypothesized that a high insulin state can contribute to cancer growth through its mitogenic properties [99]. Further adding to the complexity, certain types of functional NENs, such as glucagonoma and somatostatinoma, can induce hyperglycemia and are associated with higher rates of developing diabetes [100]. The use of therapies for NENs, such as somatostatin analogues (SSAs) and mTOR inhibitors, can also cause impaired glucose metabolism and insulin resistance [100]. Diabetes is an established risk factor for NENs, although most evidence suggests that patients with NENs and diabetes generally do not differ significantly in their outcomes in comparison to non-diabetic patients [100,101]. However, some studies have suggested that diabetes can modify the risk of metastases. Notably, in the case-control study of sporadic pancreatic endocrine tumors by Capurso et. al. [84], recent diabetes was an independent risk factor for tumor formation and correlated with a higher incidence of metastatic disease at the time of diagnosis. A correlation also exists between T2DM and an increased frequency of the pleural invasion of pulmonary carcinoids [102].

There has been some evidence that the use of metformin for glycemic control in diabetic patients improves survival in NEN patients. In an analysis of patients with advanced pancreatic NETs under treatment with everolimus and/or SSAs, the progression-free survival (PFS) of patients on metformin for diabetes was longer than both the diabetic patients not on metformin (PFS 44.2 months vs. 20.8 months, *p* < 0.0001) and the non-diabetic patients [103]. In a later post hoc analysis of the Controlled Study of Lanreotide Antiproliferative Response in Neuroendocrine Tumours (CLARINET) examining the use of Lanreotide in advanced non-functional enteropancreatic NETs with diabetes, diabetic patients receiving metformin had significantly longer progression-free survival rates in comparison to diabetic patients not receiving metformin (85.7 weeks versus 38.7 weeks, *p* = 0.009) [104]. On that basis, the METNET phase II clinical trial was specifically designed to test the role of metformin monotherapy in gastroenteropancreatic or pulmonary advanced/metastatic well-differentiated NETs [105]. This trial did not demonstrate a clinically significant anti-tumor effect, which led the authors to hypothesize that the beneficial effect of metformin is in its synergistic activity with everolimus to inhibit mTOR. Unfortunately, the clinical evidence for this is also controversial and entirely retrospective [103,105,106]. While the repurposing of a well-established medication such as metformin in cancer treatment is attractive, clearly, further studies are needed to determine whether it has a role in NEN treatment. Further examination of the correlation between obesity and NENs will need to carefully stratify patients based on history of diabetes and the use of metformin to remove possible confounding effects.

## 7. Nutrition, Obesity and NENs

A discussion of how obesity impacts NENs would not be complete without considering the significant contribution of overall nutritional status on disease course. The interpretation of how obesity impacts outcomes in NETs is confounded by the various ways in which NETs can impact gut function and metabolism by secreting hormones and small peptides similar to their normal cell counterparts, such as serotonin, gastrin, ghrelin, glucagon, somatostatin and insulin [107]. Therefore, NET patients may develop CS, diabetes, hypoglycemia or hypergastrinemia (e.g., Zollinger–Ellison syndrome) [108,109]. Examples of gastrointestinal complications with NETs include malabsorption, dysmotility, chronic diarrhea and steatorrhea [89,108,110]. These gastrointestinal complications in NETs may result from the location of the tumor within the digestive tract, secretion of hormones by functional tumors and side effects of cancer therapies [89]. Patients with NETs are also at risk for developing deficiencies of niacin, fat soluble vitamins and vitamin B12 [111,112,113,114]. These nutritional complications may be further exacerbated by the use of SSAs that inhibit the function of pancreatic enzymes and surgical treatment of NETs that alter the anatomy and function of the gastrointestinal tract [107,110,112].

The prevalence of suboptimal nutritional status among NEN patients has been demonstrated in several clinical studies. Nutritional status in cancer patients may be evaluated using screening tools such as the Subjective Global Assessment (SGA) and Nutritional Risk Screening (NRS) scores or with bioelectrical impedance analysis [88,115,116,117]. The SGA scale classifies patients as well nourished (SGA A), moderately or suspected malnourished (SGA B) and severely malnourished (SGA C) [88]. In a cross-sectional analysis performed by Barrea et. al. of 83 patients with GEP-NET [71], patients with GEP-NETs had a worse metabolic profile than the average population, were less adherent to a Mediterranean diet and consumed greater amounts of simple carbohydrates and polyunsaturated fats. This led to increased waist circumference and higher blood pressure, high levels of fasting glucose, total and LDL cholesterol, and higher triglycerides and lower HDL cholesterol [71]. Patients with progressive or stage 4 disease also had a worse metabolic profiles compared to patients with earlier stages of the disease [71]. In an international survey of 1928 patients diagnosed with NETs, a significant number of patients reported gastrointestinal side effects related to their diagnosis (48% diarrhea, 41% abdominal cramping, 21% reflux, 21% weight loss, 19% steatorrhea and 15% weight gain) and 58% reported the need for dietary changes which may negatively impact their metabolic profile [108,118]. This highlights the importance of a multi-disciplinary approach to address the complex dietary needs of NET patients [108,118].

Several studies have been undertaken to evaluate how malnutrition impacts patient outcomes in NENs. In a study that evaluated the nutritional status of patients with NENs using the SGA and NRS, patients with more advanced disease (e.g., Grade 3 NEC, patients requiring treatment with chemotherapy and patients with progressive disease) displayed higher rates of malnutrition [88]. Furthermore, malnourished patients demonstrated poorer overall survival rates (mean overall survival (OS) of 31.17 months vs. 19.94 months between SGA A and SGA B or C, with *p* < 0.001), an effect that was preserved in a subgroup analysis of different primary tumor sites, disease staging and treatment status [88]. These findings are concordant with a study by Borre et. al. [119], which demonstrated that 38% of NET patients were at nutritional risk. Using the malnutrition universal screening tool (MUST), Qureshi et. al. [120] also demonstrated that 14% of outpatients with GEP-NETs were at nutritional risk. Another study of 325 pancreatic NET patients demonstrated that patients who were underweight, defined as having a BMI of <20 at the time of diagnosis, had poorer prognoses (HR 2.5, *p* = 0.006) [90]. Independent of the screening tool used, these studies altogether demonstrate that an estimated 25% of patients with NETs may be at risk of significant malnutrition and malnutrition is ultimately linked to poorer patient outcomes [88,119,120].

As a consequence of the unique ways that NENs and their treatments may interact with a patient’s nutritional status, specific guidelines have been developed to optimize nutritional status in NENs [107,108]. Examples of important nutritional recommendations for NEN patients include supplementation with niacin, vitamin B12 and fat-soluble vitamins; screening for malnutrition; and dietary modifications when patients develop food intolerances [108,110]. In a study focused on vitamin D deficiency, Robbins et. al. [121] demonstrated that simple advice to increase vitamin D supplementation resulted in a significant improvement in vitamin D insufficiency (66% at baseline to 44.9% after 12 months), suggesting that nutritional interventions may not require significant healthcare expenditures. The targeted management of gastrointestinal side effects may also improve overall nutritional status. For example, the Telotristat Etiprate for Carcinoid Syndrome Therapy (TELECAST) phase 3 trial examined the use of Telotristat ethyl in addition to somatostatin analogues for patients who had diarrhea relating to CS, demonstrating a sustained reduction of bowel movement frequency and weight gain and improvements in nutritional status [122,123]. Future prospective studies will be necessary to optimize specific dietary interventions for NEN patients. Given the relatively good survival outcomes for patients diagnosed with NENs, the risk of developing long-term metabolic complications and cardiovascular disease should a NEN patient’s poor nutritional status not be addressed is a strong consideration, as well.

## 8. Conclusions

Obesity is a significant burden on human health, with established risks of developing multiple cancer types, although there has been evidence in support of an “obesity paradox” whereby increased BMI is thought to be a good prognostic indicator for certain cancers.

In this review article, we focused on the impact of obesity on the risk and prognosis of NENs. Epidemiological studies have demonstrated a higher incidence of NENs in individuals with obesity and MS, although obesity seems to be a good prognostic indicator for patients with NENs based on the currently available retrospective studies. Certainly, additional research is necessary to define the impact of obesity and MS on outcomes for NEN patients. In our review of the evidence, the following key areas will require further investigations:

1. Future studies should be designed to deconvolute the individual contribution of visceral adiposity as an independent prognostic factor for NENs while controlling for individual differences in metabolic profile, diabetes, nutritional status and diet, and the co-existence of cachexia and cancer treatment.
2. Future studies are necessary to evaluate whether the impact of obesity on the prognosis of NENs can be extrapolated to tumors outside of the gastrointestinal system, as well as to neuroendocrine carcinoma.
3. A prospective study examining specific nutritional interventions and their impact on both survival and patient-reported outcomes will serve to evaluate their impacts and define treatment protocols. Such studies will need to control for known factors in the risk and prognosis of NENs. A standardized tool for malnutrition should be performed before and after the intervention.
4. The measure of both BMI and more advanced metrics of visceral obesity should be compared to determine the validity of BMI as a metric in similar studies of obesity and cancer outcomes.

Given the relative paucity of data in support of the obesity paradox in NENs, the known nutritional complications relating to NENs and the overall negative health outcomes associated with obesity, it would be premature to make a recommendation targeting a specific BMI for patients with NENs. However, a review of the current literature highlights the importance of weight and nutritional assessment as regular components of the evaluation of patients with NENs, as well as a multi-disciplinary approach to the management of GI side effects, weight loss, malnutrition and cancer cachexia.
